# The effect of casirivimab with imdevimab on disease progression in nonsevere COVID‐19 patients in a single hospital in Japan

**DOI:** 10.1002/jgf2.516

**Published:** 2021-12-19

**Authors:** Junpei Komagamine, Taku Yabuki, Satsuki Yoshihara, Nao Tanaka

**Affiliations:** ^1^ Department of Internal Medicine National Hospital Organization Tochigi Medical Center Utsunomiya Japan

**Keywords:** casirivimab, COVID‐19, imdevimab

## Abstract

**Background:**

Recent randomized trials have revealed that neutralizing monoclonal antibodies can reduce disease progression in mild–moderate COVID‐19 patients. However, no studies have investigated the effect of neutralizing monoclonal antibodies on clinical outcomes in Japan.

**Methods:**

A single‐center retrospective and prospective cohort study was conducted. All consecutive febrile nonsevere COVID‐19 patients with at least one risk factor were included. The primary outcome was progression to severe COVID‐19. Severe COVID‐19 cases were defined as patients requiring oxygen therapy and dexamethasone. The differences in the primary outcomes between the patients who were treated with casirivimab with imdevimab (treatment group) and those who were not (control group) were compared using the chi‐squared test.

**Results:**

A total of 128 patients were included. Of those, the mean age was 53.6 years old (SD 9.9), and 52 (40.6%) were women. Fifty‐three patients were treated with casirivimab with imdevimab, and 75 patients were given the standard treatment only. The primary outcome occurred in eight (15.1%) of the 53 patients in the treatment group and 33 (44.0%) of the 75 patients in the control group (odd ratio [OR] 0.23, 95% CI 0.09 to 0.55). The multivariate analysis revealed that the use of casirivimab with imdevimab (OR 0.21, 95% CI 0.08 to 0.54) was the only independent risk factor associated with progression to severe COVID‐19. No patients died during hospitalization in either group.

**Conclusion:**

Similar to other countries, casirivimab with imdevimab significantly reduced disease progression in early nonsevere COVID‐19 patients with fever and risk factors in Japan.

## Graphical abstract

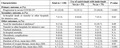

## 1 INTRODUCTION

Since the first case of severe acute respiratory syndrome coronavirus 2 (SARS‐CoV‐2) was documented in December 2019 in China,^1^ several treatments for coronavirus disease 2019 (COVID‐19) have been developed. Dexamethasone and interleukin (IL)‐6 receptor antagonists have been proven to decrease the mortality of severe COVID‐19 patients.^2^ However, there are few effective therapies for nonsevere COVID‐19 patients. Therefore, treatments to prevent disease progression in nonsevere COVID‐19 patients are urgently needed.

There is increasing interest in the use of neutralizing monoclonal antibodies (mAbs) to prevent disease progression in nonsevere COVID‐19 patients, including asymptomatic patients.^3^, ^4^, ^5^, ^6^ Neutralizing mAbs can bind to the SARS‐CoV‐2 spike protein and neutralize the SARS‐CoV‐2 virus in infected patients.^3^ Recent randomized controlled trials have reported that neutralizing mAbs, such as casirivimab with imdevimab, for early nonsevere COVID‐19 patients could reduce the rates of hospitalization and death by 70%.^6^, ^7^, ^8^ Nonetheless, there are few data about the efficacy of mAbs in real‐world practice. Moreover, no Japanese studies have investigated the effect of casirivimab with imdevimab on the clinical outcomes of nonsevere COVID‐19 patients. Given that the difference in the mortality rate due to COVID‐19 between Japan and other countries has been reported,^9^ the impact of casirivimab with imdevimab on the clinical outcomes in Japan might be different from that of other countries. Therefore, the aim of our study was to determine the effects of casirivimab with imdevimab on disease progression in early nonsevere COVID‐19 patients in a Japanese hospital.

## 2 METHODS

### 2.1 Study design and setting

A retrospective (from July 20 to July 31, 2021) and prospective (from August 1 to September 9, 2021) cohort study was conducted using the electronic medical records of our general community hospital in Japan. Our hospital provides acute care and is designated to provide care for COVID‐19 patients in this region, which has a population of approximately 0.5 million. More than 70% of SARS‐CoV‐2 viruses identified in this area during the study period were Delta variants. Approximately one‐fifth of all COVID‐19 patients in this region were admitted to our hospital during the study period. The disease severity was mild to severe in most of the COVID‐19 patients admitted to our hospital. If intensive care was needed for the care of the COVID‐19 patients, the patients were transferred to other tertiary hospitals. Treatment of COVID‐19 in our hospital was based on the WHO guidelines.^2^ This research was approved by the Medical Ethical Committee of our hospital and was conducted in accordance with the Declaration of Helsinki. The need for individual informed consent was formally waived by the institutional Medical Ethics Committee because we collected de‐identified data without contacting the patients. However, per the Japanese Ethical Guidelines, we displayed an opt‐out statement in the waiting room and on the webpage of the hospital to inform patients about the study and to provide the opportunity for patients to decline the use of their data.

### 2.2 Screening of the patients and the inclusion and exclusion criteria

The data of COVID‐19 patients hospitalized in our hospital from July 20, 2021, to September 9, 2021, were used. To minimize selection bias, all consecutive COVID‐19 patients hospitalized during the study period were screened. This date was chosen because casirivimab with imdevimab became available on July 20, 2021 in our region. Patients who met all the following criteria were included. (1) The patient's age was 18 years old or older. (2) COVID‐19 was diagnosed based on nucleic acid tests or antigen tests. (3) The duration of COVID‐19 symptoms was less than 7 days. (4) The patient had at least one risk factor for severe COVID‐19. Based on the Japanese pharmaceutical reference,^10^ the risk factors were diabetes, cardiovascular disease (including hypertension), age greater than 50 years old, obesity (body mass index ≥30), chronic lung disease (including asthma), chronic liver disease, chronic kidney disease, and immunosuppressive status. (5) Fever (≥37.5°C) associated with COVID‐19 was documented within 24 h before admission or during hospitalization. Based on recent trials, 20 to 30 patients were required to receive mAb treatment to prevent one nonsevere COVID‐19 patient from hospitalization or death. Given the high cost of neutralizing mAbs, we believe that higher‐risk patients should be selected for mAb treatment. Based on our hospital data, approximately half of COVID‐19 patients who were febrile and had at least one risk factor progressed to severe COVID‐19, requiring oxygen therapy and dexamethasone. Therefore, we added fever as an inclusion criterion. Patients whose symptoms had started more than 14 days after the second COVID‐19 vaccination were excluded. Patients who required oxygen therapy due to persistent hypoxia at admission were also excluded. In routine practice, we contacted the eligible patients and asked if they wanted to receive casirivimab with imdevimab. If they chose to receive this therapy, casirivimab 600 mg with imdevimab 600 mg was administered intravenously according to the Japanese pharmaceutical reference.^10^ Patients who received casirivimab with imdevimab were included as the treatment group, while patients who did not receive casirivimab with imdevimab for any reason were included as the control group.

### 2.3 Data collection and outcome measures

Information on patient age, gender, the Charlson Comorbidity Index score,^11^ past medical history, vital signs, clinical symptoms, and the presence of pneumonia at admission was retrospectively extracted from the electronic medical records. Pneumonia was diagnosed if the patients had new infiltration on chest images or had crackles on the physical examination at admission.

The primary outcome was progression to severe COVID‐19. Based on the WHO guidelines,^2^ severe COVID‐19 cases were defined as patients requiring oxygen therapy and dexamethasone. The secondary outcomes were a composite outcome that included critical care needs and in‐hospital death. Other outcomes included the duration of fever and the duration of hospital stay. The duration of fever was calculated from the hospital day when casirivimab with imdevimab was considered treatment for the patients to the first day when the patients were afebrile.

### 2.4 Statistical analysis

Based on recent randomized controlled trials,^8^ we assumed that casirivimab with imdevimab could reduce the proportion of patients who met the primary outcome by 70%. In the study period, approximately 50% of patients who met the inclusion criteria progressed to severe COVID‐19 after receiving the usual care in our hospital. Therefore, we estimated that a sample size of approximately 50 patients per group would provide an 80% chance of detecting a significant difference between the treatment and control groups. However, we did not perform the sample size calculation for logistic regression before starting the study. Therefore, this might limit our multivariate analysis.

Descriptive statistics were used to report the characteristics of the included patients. For the primary and secondary outcomes, patients who received casirivimab with imdevimab (treatment group) were compared with patients who did not receive casirivimab with imdevimab (control group) using the chi‐squared test or Student's *t* test. To determine the risk factors associated with progression to severe COVID‐19, a multivariate analysis using binary logistic regression was conducted. The association between the progression to severe COVID‐19 and selected variables was investigated. The variables that were adjusted in the model included COVID‐19 vaccination status, gender, presence of pneumonia at admission, number of risk factors, and days from symptom onset. Male gender and the presence of pneumonia at admission were included because they were also reported to be associated with poor prognosis of COVID‐19.^12^, ^13^ Even only one COVID‐19 vaccination could reduce the risk of progression of COVID‐19.^14^ Therefore, the vaccination status was included. It is possible that the effect of casirivimab with imdevimab might depend on the timing from symptom onset.^8^, ^15^ Therefore, days from symptom onset were also included. We did not make the group continuous variables to create new categorical variables, and two variables (number of risk factors and days from symptom onset) were handled as continuous variables. There were no missing data for the variables used in the multivariate analysis. The level of statistical significance was set at 5%. These analyses were performed by using Stata version 15 (LightStone).

## 3 RESULTS

During the study period, 337 COVID‐19 patients were hospitalized in our hospital. Of those, 128 patients who met the inclusion criteria were included in the final analysis. Of all of the patients, the mean age was 53.6 years (SD 9.9), 52 (40.6%) were women, and most were Japanese (Table 1 and Table S1). The mean number of risk factors associated with progression to severe COVID‐19 was 1.7 (0.8), and the mean time from symptom onset was 4.4 days (SD 1.5). Thirty‐nine patients (30.5%) were diagnosed with pneumonia at admission. Of all of the patients, 121 (94.5%) recovered and were discharged (78 were discharged to home, and 43 were discharged to the hotel for isolation). The mean follow‐up duration was 8.8 days (SD 6.9).

**TABLE 1:** Characteristics of the 128 consecutive febrile patients with non‐severe COVID‐19

Characteristics	Total (*n* = 128)	Use of casirivimab with imdevimab	
		Yes (*n* = 53)	No (*n* = 75)
Median age (IQR), years	53 (48 to 59)	51 (47 to 58)	54 (50 to 59)
Female sex, *n* (%)	52 (40.6)	27 (50.9)	25 (33.3)
Race, *n* (%)			
Japanese	121 (94.5)	48 (90.6)	73 (97.3)
Others	7 (5.5)	5 (9.4)	2 (2.7)
At least one COVID‐19 vaccination^a^, *n* (%)	27 (21.1)	14 (26.4)	13 (17.3)
Median Body Mass Index (IQR)	25.6 (23.1 to 29.3)	26.2 (22.7 to 31.2)	25.2 (23.2 to 28.6)
Median Charlson Comorbidity Index (IQR)	0 (0 to 1)	0 (0 to 1)	0 (0 to 0)
Risk factors			
Number of risk factors (SD)	1.7 (0.8)	1.8 (0.9)	1.6 (0.7)
BMI ≧ 30, *n* (%)	30 (23.4)	16 (30.2)	14 (18.7)
Aged more than 50 years old, *n* (%)	90 (70.3)	34 (64.2)	56 (74.7)
Cardiovascular disease, *n* (%)	50 (39.1)	24 (45.3)	26 (34.7)
Chronic kidney disease, *n* (%)	1 (0.8)	1 (1.9)	0 (0.0)
Diabetes mellitus, *n* (%)	22 (17.2)	8 (15.1)	14 (18.7)
Chronic lung disease, *n* (%)	12 (9.4)	7 (13.2)	5 (6.7)
Chronic liver disease, *n* (%)	3 (2.3)	2 (3.8)	1 (1.3)
Immuno‐compromised status, *n* (%)	5 (3.9)	4 (7.6)	1 (1.3)
Median days from symptom onset (IQR)	5 (3 to 6)	5 (3 to 5)	5 (3 to 6)
Pneumonia at admission^b^, *n* (%)	39 (30.5)	15 (28.3)	24 (32.0)

^a^ This value excluded the patients who were admitted more than 14 days after the second COVID‐19 vaccination.

^b^ This included patients who were diagnosed with pneumonia by physical examination or chest imaging tests at admission.

Casirivimab with imdevimab was unavailable for 42 patients in time for treatment, and the principal physicians were unaware of an indication for casirivimab with imdevimab for 11 patients. Twelve patients preferred to not have treatment with casirivimab with imdevimab. Because the patients already tended to recover at admission, casirivimab with imdevimab was judged to be unnecessary by the principal physicians for 10 patients. Therefore, a total of 75 patients (58.6%) did not receive casirivimab with imdevimab and were included as the control group. The remaining 53 patients who received casirivimab with imdevimab were included as the treatment group.

The primary outcome occurred in 41 (32.0%) patients (Table 2). The progression to severe COVID‐19 in the treatment group was significantly less than that in the control group (15.1% vs. 44.0%, odds ratio [OR] 0.23; 95% CI 0.09 to 0.55). For the secondary outcomes, the need for critical care and in‐hospital death were significantly less likely to occur in the treatment group than in the control group (0.0% vs. 9.3%). No patients died during hospitalization in either group. No anaphylaxis was documented in the treatment group. The duration of fever was significantly shorter in the treatment group than in the control group (5.4 days vs. 7.5 days). The duration of hospital stay was also significantly shorter in the treatment group than in the control group (5.4 days vs. 7.6 days). The multivariable analysis revealed that the use of casirivimab with imdevimab (OR 0.21, 95% CI 0.08 to 0.54) was the only independent risk factor associated with progression to severe COVID‐19 (Table 3).

**TABLE 2:** The primary and secondary outcomes among COVID‐19 patients who received casirivimab with imdevimab and those who did not

Characteristics	Total (*n* = 128)	Use of casirivimab with imdevimab		*p*‐Value
		Yes (*n* = 53)	No (*n* = 75)	
**Primary outcome, *n* (%)**				
Progression to severe COVID‐19^a^	41 (32.0)	8 (15.1)	33 (44.0)	<0.001
**Secondary outcome, *n* (%)**				
In‐hospital death or transfer to other hospitals for intensive care	7 (5.5)	0 (0.0)	7 (9.3)	0.02
**Other outcomes, *n* (%)**				
Need for interleukin‐6 inhibitors^b^	9 (7.0)	1 (1.9)	8 (10.7)	0.09
Transfer to other hospitals for intensive care	7 (5.5)	0 (0.0)	7 (9.3)	0.02
Tracheal intubation	2 (1.6)	0 (0.0)	2 (2.7)	0.23
In‐hospital mortality	0 (0.0)	0 (0.0)	0 (0.0)	N.A.
Thrombotic complications	0 (0.0)	0 (0.0)	0 (0.0)	N.A.
Anaphylaxis	0 (0.0)	0 (0.0)	0 (0.0)	N.A.
Duration of fever, mean days (SD)	6.6 (2.6)	5.4 (1.7)	7.5 (2.8)	<0.001
Duration of oxygen therapy, mean days (SD)	1.2 (2.2)	0.7 (1.9)	1.6 (2.3)	0.03
Duration of hospital stay, mean days (SD)	6.7 (3.2)	5.4 (3.0)	7.6 (3.1)	<0.001

^a^ Cases of severe COVID‐19 were defined if the patient required both oxygen supplemental therapy and dexamethasone.

^b^ Interleukin‐6 inhibitors were used for the COVID‐19 patients who did not respond to dexamethasone therapy.

**TABLE 3:** Univariable and multivariable analyses^a^ for the factors associated with the progression to severe COVID‐19

Variables	Progression to severe COVID‐19	
	Unadjusted OR (95% CI)	Adjusted OR^b^ (95% CI)
Use of casirivimab with imdevimab	0.23 (0.09 to 0.55)*	0.21 (0.08 to 0.54)*
Any COVID‐19 vaccination^c^	0.87 (0.34 to 2.19)	1.18 (0.42 to 3.31)
Women	0.78 (0.36 to 1.67)	0.86 (0.37 to 2.01)
Pneumonia at admission	2.09 (0.95 to 4.60)	1.92 (0.78 to 4.68)
Number of risk factors^d^, ^e^	1.10 (0.70 to 1.71)	1.27 (0.76 to 2.14)
Days from symptom onset^e^	1.16 (0.89 to 1.51)	1.13 (0.84 to 1.51)

^a^ The threshold for statistical significance was set at *p* < 0.05. Asterisks indicate a significant association between the selected variables and progression to severe COVID‐19.

^b^ The variables that were adjusted in the model included use of casirivimab with imdevimab, COVID‐19 vaccination status, sex, presence of pneumonia at admission, number of risk factors, and days from symptom onset.

^c^ This value excluded patients who were admitted more than 14 days after the second COVID‐19 vaccination.

^d^ This value included patients with an age greater than 50 years old, obesity, diabetes, cardiovascular disease including hypertension, chronic lung disease including asthma, chronic liver disease, chronic kidney disease, and immunocompromised status.

^e^ Continuous varibles were used.

## 4 DISCUSSION

Our findings showed that casirivimab with imdevimab could reduce disease progression in nonsevere COVID‐19 patients by approximately 80% in real‐world practice. This result is consistent with that of recent randomized controlled trials,^6^, ^7^, ^8^ which have shown that neutralizing mAb therapy reduced disease progression in nonsevere COVID‐19 by 70%. This implies that neutralizing mAb therapy can also prevent disease progression in nonsevere COVID‐19 patients in Japan, similar to other countries.

To improve the cost performance of casirivimab with imdevimab, we included only febrile patients who also had at least one risk factor associated with progression to severe COVID‐19. Based on the results of the present study, the number needed to treat to prevent disease progression in nonsevere febrile COVID‐19 patients was approximately 3.6. This rate is much lower than that of recent randomized controlled trials.^6^, ^7^, ^8^ Given the high cost and limited supply of casirivimab with imdevimab, our strategy to select and treat higher‐risk groups with these monoclonal antibodies would be important. Further studies are warranted to identify COVID‐19 patients who would benefit more from neutralizing mAb therapy.

Several limitations should be mentioned. First, the limitations of our study include the observational study design and the small sample size. However, our results are consistent with those of past randomized controlled trials.^6^, ^7^, ^8^ Second, we did not obtain data on most of the patients after discharge. Therefore, the long‐term effects of casirivimab with imdevimab remain uncertain. Third, the majority of the SARS‐CoV‐2 viruses in our region during the study period were identified as Delta variants. Therefore, the effectiveness of casirivimab with imdevimab for other variants of the SARS‐CoV‐2 virus remains uncertain. Finally, the occurrence of the primary outcome was less frequent than we expected. Given that at least 10 events per variable were desirable to maintain the validity of the multivariate analysis,^16^ use of four variables was appropriate. Therefore, our findings of the multivariate analysis might be limited. However, a posthoc multivariate analysis using four variables showed a similar result for the effect of casirivimab with imdevimab on the primary outcome (Table S2).

In conclusion, casirivimab with imdevimab significantly reduced the progression to severe COVID‐19 in early nonsevere COVID‐19 patients in Japan who had fever and risk factors, which is similar to other countries. Given the high cost and limited supply of casirivimab with imdevimab, a strategy to select and treat higher‐risk groups with these monoclonal antibodies might be needed.

## CONFLICT OF INTEREST

The authors have stated explicitly that there are no conflicts of interest in connection with this article.

## Supporting information

Table S1‐S2
