# Supplementary material for: The effect of casirivimab with imdevimab on disease progression in nonsevere COVID‐19 patients in a single hospital in Japan
Source: J Gen Fam Med. 2021 Dec 19;23(3):158–63. doi: 10.1002/jgf2.516 (PMC9062551; doi:10.1002/jgf2.516)
Supplement: Supplementary file 1 — Table S1‐S2 [file JGF2-23-158-s001.pdf]

Table S1. Characteristics and treatments of the 128 consecutive febrile patients with non-severe COVID-19.

**Characteristics**	**Total (n = 128)**	**Use of casirivimab with imdevimab**	
		**Yes (n = 53)**	**No (n = 75)**
Age, years			
Mean (SD)	53.6 (9.9)	53.2 (10.2)	54.0 (9.7)
Median (IQR)	53 (48 to 59)	51 (47 to 58)	54 (50 to 59)
Female sex, n (%)	52 (40.6)	27 (50.9)	25 (33.3)
Race, n (%)			
Japanese	121 (94.5)	48 (90.6)	73 (97.3)
Others	7 (5.5)	5 (9.4)	2 (2.7)
Current smoker, n (%)	25 (19.5)	8 (15.1)	17 (22.7)
Regular alcohol drinker, n (%)	36 (28.1)	14 (26.4)	22 (29.3)
At least one COVID-19 vaccination[a], n (%)	27 (21.1)	14 (26.4)	13 (17.3)
Body Mass Index			
Mean (SD)	26.5 (5.2)	27.3 (6.4)	26.0 (4.2)
Median (IQR)	25.6 (23.1 to 29.3)	26.2 (22.7 to 31.2)	25.2 (23.2 to 28.6)
Charlson Comorbidity Index			
Mean (SD)	0.4 (0.6)	0.5 (0.7)	0.3 (0.6)
Median (IQR)	0 (0 to 1)	0 (0 to 1)	0 (0 to 0)
Medical history, n (%)			
Stroke	7 (5.5)	4 (7.6)	3 (4.0)
Ischemic heart disease	1 (0.8)	1 (1.9)	0 (0.0)
Dementia	1 (0.8)	1 (1.9)	0 (0.0)
COPD or asthma	11 (8.6)	7 (13.2)	4 (5.3)
Heart failure	1 (0.8)	0 (0.0)	1 (1.3)
Hypertension	46 (35.9)	20 (37.7)	26 (34.7)
Dyslipidemia	36 (28.1)	19 (35.9)	17 (22.7)
Atrial fibrillation	2 (1.6)	0 (0.0)	2 (2.7)
Venous thrombosis	0 (0.0)	0 (0.0)	0 (0.0)
Regularly used medications			
Total number (SD)	2.0 (2.4)	2.3 (2.6)	1.7 (2.3)
Antiplatelet drugs, n (%)	8 (6.3)	5 (9.4)	3 (4.0)
Anticoagulant drugs, n (%)	3 (2.3)	0 (0.0)	3 (4.0)
Oral corticosteroid, n (%)	4 (3.1)	3 (5.7)	1 (1.3)
Corticosteroid inhaler, n (%)	4 (3.1)	3 (5.7)	1 (1.3)

Risk factors			
Number of risk factors (SD)	1.7 (0.8)	1.8 (0.9)	1.6 (0.7)
BMI ≧ 30, n (%)	30 (23.4)	16 (30.2)	14 (18.7)
Aged more than 50 years old, n (%)	90 (70.3)	34 (64.2)	56 (74.7)
Cardiovascular disease, n (%)	50 (39.1)	24 (45.3)	26 (34.7)
Chronic kidney disease, n (%)	1 (0.8)	1 (1.9)	0 (0.0)
Diabetes mellitus, n (%)	22 (17.2)	8 (15.1)	14 (18.7)
Chronic lung disease, n (%)	12 (9.4)	7 (13.2)	5 (6.7)
Chronic liver disease, n (%)	3 (2.3)	2 (3.8)	1 (1.3)
Immuno-compromised status, n (%)	5 (3.9)	4 (7.6)	1 (1.3)
Symptoms at admission, n (%)			
Fever[b]	128 (100.0)	53 (100.0)	75 (100.0)
Cough	87 (68.0)	36 (67.9)	51 (68.0)
Malaise	91 (71.1)	39 (73.6)	52 (69.3)
Headache	42 (32.8)	16 (30.2)	26 (34.7)
Taste dysfunction	24 (18.8)	8 (15.1)	16 (21.3)
Olfactory dysfunction	22 (17.2)	10 (18.9)	12 (16.0)
Diarrhea	25 (19.5)	12 (22.6)	13 (17.3)
Dyspnea	12 (9.4)	4 (7.6)	8 (10.7)
Days from symptom onset			
Mean (SD)	4.4 (1.5)	4.3 (1.5)	4.5 (1.5)
Median (IQR)	5 (3 to 6)	5 (3 to 5)	5 (3 to 6)
Body temperature[c]			
Mean (SD)	38.5 (0.6)	38.5 (0.6)	38.5 (0.6)
Median (IQR)	38.4 (38.0 to 38.9)	38.5 (38.0 to 39.0)	38.3 (38.0 to 38.9)
Saturated oxygen[d], % (room air)			
Mean (SD)	95.3 (1.6)	95.3 (1.8)	95.3 (1.5)
Median (IQR)	95 (94 to 97)	95 (94 to 97)	95 (95 to 96)
Pneumonia at admission[e], n (%)	39 (30.5)	15 (28.3)	24 (32.0)
Treatment during hospitalization, n (%)			
Any heparin use	0 (0.0)	0 (0.0)	0 (0.0)
Any oral anticoagulants	0 (0.0)	0 (0.0)	0 (0.0)
Any antimicrobial drugs	0 (0.0)	0 (0.0)	1 (1.3)
Antiviral agents including remdesivir	0 (0.0)	0 (0.0)	0 (0.0)
Dexamethasone	41 (32.0)	8 (15.1)	33 (44.0)
Tocilizumab	9 (7.0)	1 (1.9)	8 (10.7)

Recovery to discharge to home or hotels[f] for isolation, n (%)	121 (94.5)	53 (100.0)	68 (90.7)

[a]This value excluded the patients who were admitted more than 14 days after the second COVID-19 vaccination.

[b]Fever was defined as a temperature more than 37.5 degrees Celsius using any body site.

[c]The maximum body temperature within 24 hours before study inclusion.

[d]The lowest saturated oxygen within 24 hours before study inclusion.

[e]This included patients who were diagnosed with pneumonia by physical examination or chest imaging tests at admission.

[f]When the COVID-19 patients recovered but needed isolation, they were transferred to the hotel for isolation in our region of Japan.

Table S2. Multivariable analysis[a] for the factors associated with the progression to severe COVID-19.

**Variables**	**Adjusted odds ratio[b] (95% CI)**
Use of casirivimab with imdevimab	0.21 (0.08 to 0.52)*
Any COVID-19 vaccination[c]	1.12 (0.40 to 3.08)
Pneumonia at admission	2.06 (0.88 to 4.80)
Number of risk factors[d,e]	1.22 (0.74 to 2.02)

[a]The threshold for statistical significance was set at $p < 0.05$. Asterisks indicate a significant association between the selected variables and progression to severe COVID-19.

[b]The variables that were adjusted in the model included use of casirivimab with imdevimab, COVID-19 vaccination status, presence of pneumonia at admission, and number of risk factors.

[c]This value excluded patients who were admitted more than 14 days after the second COVID-19 vaccination.

[d]This value included patients with an age greater than 50 years old, obesity, diabetes, cardiovascular disease including hypertension, chronic lung disease including asthma, chronic liver disease, chronic kidney disease, and immunocompromised status.

[e]Continuous varibles were used.
